# Editorial: Anxiety Disorders in Childhood and Adolescence: Psychopathology, Assessment, and Treatment

**DOI:** 10.3389/fpsyg.2022.930299

**Published:** 2022-06-20

**Authors:** Francisco J. Méndez, Mireia Orgilés, José P. Espada, José M. García-Fernández, Cecilia A. Essau

**Affiliations:** ^1^Department of Personality, Assessment, and Psychological Treatment, Universidad of Murcia, Murcia, Spain; ^2^Department of Health Psychology, Miguel Hernández University, Elche, Spain; ^3^Department of Developmental Psychology and Teaching, University of Alicante, Alicante, Spain; ^4^Department of Psychology, University of Roehampton, London, United Kingdom

**Keywords:** anxiety, children, psychopathology, psychological treatment, clinical psychology, adolescents, assessment

Fear and anxiety share a pattern of psychophysiological (palmar sweating, tachycardia, hyperventilation, muscle tension, etc.), cognitive (worry, expectation of harm, negative evaluation of personal coping skills, perceptual distortion, etc.), and motor (trembling, stuttering, escape, avoidance, etc.) responses to potentially dangerous situations. In fears, external stimuli, the present situation and motor responses predominate, whereas in anxiety internal stimuli, anticipation of the situation and cognitive responses prevail. Thus, in fears the child easily identifies the threat, for example the dog or the storm, and reacts by escaping from the situation and in anxiety the child may not recognize the source that provokes it, for example his or her competence in studying, and responds with worry. Perhaps because in fear the greater weight falls on motor responses and in anxiety on cognitive responses, specific phobia appears at earlier ages than generalized anxiety disorder.

Fear and anxiety are present in childhood because of their adaptive role: fear of academic failure drives the schoolchild to study, anxiety about the negative evaluation of the audience drives the speaker to prepare the speech, fear of injury drives the motorcyclist to put on the helmet, etc. However, when the child's reaction is disproportionate, either because the feared situation is harmless, e.g. darkness, or because it involves a certain risk, e.g. an exam with the possibility of failing, the child responds exaggeratedly and goes blank, then the fear is called phobia and the anxiety, anxiety disorder.

Anxiety disorders are among the most frequent disorders in childhood and adolescence, and their prevalence is estimated between 7 and 12% (Canals et al., 2019; Ghandour et al., 2019). They present a high comorbidity among them, higher in child and adolescent population (Curry and March, 2004), and tend to persist into adulthood (Beidel and Turner, 2007); it is estimated that 75% of adult anxiety disorders started in childhood, with a mean age of onset between 8 and 12 years (Kessler et al., 2005).

The negative impact of anxiety disorders on personal and social domains is high and they are among the top ten causes of mortality in adolescence, especially in girls (World Health Organization, 2014). Its negative repercussions include poverty of interpersonal relationships, poor academic performance and personal difficulties.

The genesis and maintenance is the result of the combined action of different factors: (a) genetic: Gregory and Eley (2007) consider that numerous genes are involved resulting in a high heritability, for example 73% in separation anxiety disorder and 61% in agoraphobia; (b) personal: behavioral inhibition (Rapee et al., 2009), negative affectivity (neuroticism) (Espada et al., 2021), selective attention and threat overvaluation (Hadwin et al., 2006) play an important role in anxiety disorders in general, fear of negative evaluation in social anxiety disorder (Morales et al., 2016), anxiety sensitivity in panic disorder (Sandín et al., 2012) and harm avoidance in generalized anxiety disorder (American Psychiatric Association, 2013); (c) familial: marital conflicts (Yap et al., 2014) and overprotective upbringing (Orgilés et al., 2018) influence the etiology of various anxiety disorders; (d) environmental: stressful life events can act as triggers (Allen et al., 2008), for example traumatic separation from the attachment figure.

The main objective of this Research Topic is to disseminate advances in the field of psychopathology, assessment and treatment of anxiety disorders in childhood and adolescence. The collection gathers a wide range of articles carried out in different areas of the world. Perhaps the nationality of the editors has influenced the fact that two thirds of articles come from Europe and one third from Spain; the geographical distribution is as follows: Europe 66.7% (Spain 33.3%, Norway 14.3%, Germany 9.5%, Holland 4.8%, Portugal 4.8%); North America 14.3% (USA 9%, Canada 4.8%), Asia 9.6% (Japan 4.8%, Saudi Arabia 4.8%) and Australia 9.5%. Most belong to the field of psychopathology (47.7%), followed by assessment (28.5%) and treatment (23.8%). The largest number of articles deal with internalized or emotional problems, which may also include depression and other disorders (38%), and the rest with social anxiety disorder (19%), anxiety disorders as a whole (14.3%), selective mutism (9.5%), related problems such as post-traumatic stress disorder or school rejection (9.5%), separation anxiety disorder (4.8%) and specific phobia (4.8%). Probably because most cases of panic disorder, agoraphobia and generalized anxiety start at older ages there is no article dedicated to these disorders. Participants in the studies have been children (47.7%), adolescents (33.3%), combined samples of children and adolescents and, exceptionally, parents or other adults (19%). Most of the articles are empirical studies (81%) and the rest are reviews or theoretical proposals (19%).

The articles reveal multiple research interests. We will present a synthesis of them grouped by field of study.

## Psychopathology

Articles deal with characterization, classification, epidemiology, risk and protective factors, comorbidity, and other aspects of internalizing problems, anxiety disorders, and related disorders.

Kearney and Rede's interesting review of selective mutism: history of its conceptualization, empirical clinical profiles, differences and similarities with other disorders, assessment and treatment, leads the authors to propose its classification as a neurodevelopmental disorder rather than as an anxiety disorder, based on the multifaceted and heterogeneous nature of the disorder. Muris et al. further along these lines propose the relationship of selective mutism with social anxiety, autistic characteristics and behavioral inhibition. Social anxiety disorder is the most common comorbid condition and behavioral inhibition is considered a risk factor for selective mutism, whereas it is very original to consider the association with autistic traits and the study contributes to clarify the nature of the disorder.

The higher prevalence of specific phobias in girls has been explained by a combination of biological and cultural factors. Gerdes et al. test the influence of mothers' gender stereotypes on their daughters' fear of snakes; indirectly the study also provides data in favor of the hypothesis of emotional contagion of fears.

Two articles are devoted to social anxiety. Ballespí et al. carry out a pioneering study on the moderating role of self and other people's mentalization on the relationship between social anxiety and personal and social deterioration. The findings highlight the importance of emotional self-awareness in the prevention and treatment of excessive social anxiety. In Young-HUNT3, the third wave of the Trøndelag Health Study, Jystad et al. study the occurrence, sociodemographic characteristics, and psychiatric comorbidities of social anxiety disorder. The clarification of these issues is relevant, for example prevalence estimates range from 0.5 to 7%, with important variations between areas, the disorder is more common in the West than in the East and, among Western countries, more common in USA than in Europe.

Three articles focus on the personal and family factors of internalizing problems and emotional disorders. The study by Raposo and Francisco on the relationship of personal wellbeing, emotional regulation and family environment with internalizing problems and the differences between high- and low-risk adolescents provides insight into the influence of these personal and family variables and serves as a guide to selective prevention interventions to improve the psychological adjustment of adolescents. The research by Sandín et al. is extremely interesting because it analyzes the incremental validity of coronavirus fears and transdiagnostic variables in predicting the severity of anxiety and depressive symptoms, supporting the need to take transdiagnostic vulnerability and protective factors into consideration in the treatment of emotional disorders. Chevalier et al. wonder whether the reflective functioning of mothers and children and adolescents is associated with anxiety and internalizing problems in minors and whether it predicts them beyond the effect of attachment. In view of the results, they conclude that psychological treatments should take into account reflective functioning to help children and adolescents to interpret anxiety symptoms and thus reduce them.

Finally, there are two articles on related problems. Gonzálvez et al. using latent profile analysis identify five affective profiles and examine their relationship with four types of school refusal behavior. The results of the study are very useful because they help professionals to develop programs to promote the most adaptive affective profiles and prevent school refusal. In the DSM-IV-TR, traumatic stress disorder was included in the anxiety disorders because of the significant increase in arousal; the close relationship of the disorder with anxiety justifies the inclusion of the study by Prieto et al. in this collection. The analysis of the impact more than 20 years after having suffered a terrorist attack in childhood, adolescence or adulthood is very novel and sheds light on whether traumatic experiences are processed differently at different ages.

## Assessment

Several instruments for collecting information on emotional problems and anxiety are presented. Piqueras et al. develop a new web-based screening questionnaire for children and adolescents for symptoms of a wide range of emotional disorders: separation anxiety, specific phobia, social anxiety, panic disorder/agoraphobia, global distress, obsessions and compulsions, posttraumatic stress, major depression, persistent depression, and suicidality. The article is highly topical because the COVID-19 pandemic has forced the administration of brief screening measures online. Lippert et al., in an effort to overcome the antagonism between the idiographic: individualized hierarchies, and nomothetic: standardized questionnaires, approaches to assess anxiety in childhood and adolescence, created the Anxiety and Avoidance Scale for Children (AVAC), an accurate and personalized instrument that takes into account individual differences in separation anxiety disorder, specific phobia and social anxiety disorder.

Other instruments focus on variables related to emotional problems. Burgdorf and Szabó validated with mothers the English adaptation of the Interpersonal Mindfulness in Parenting (IMP), an instrument that predicts internalizing problems in children and adolescents, which is useful for assessing mindful parenting programs, an educational strategy beneficial for parents and children. Expressed emotion is a construct used to describe family relationships with the member presenting with an anxiety or stress disorder, which has been shown to influence treatment outcomes. Muela-Martinez et al. conducted the Validation of the Structured Interview for the Assessment of Expressed Emotion (E5), a brief, valid and reliable measure to assess expressed emotion in parents of adolescent children.

A specific measure of separation anxiety in childhood is the Children's Separation Anxiety Scale (CSAS-P), validated in this Collection by Méndez et al. in the multisource assessment framework. The parent version complements the child's self-report and allows the degree of agreement between parents and child to be obtained. The novelty is the inclusion of the subscale Calm before separation from the attachment figure, a protective factor of the disorder. Finally, halfway between assessment and treatment, Rasmussen et al. assessed the psychometric properties and applicability of the Competence and Adherence Scale for Cognitive Behavioral Therapy (CAS CBT), using video recordings of sessions of the EMOTION: Kids Coping with Anxiety and Depression program, a transdiagnostic preventive intervention, with a cognitive-behavioral orientation, aimed at children with anxious and depressive symptoms.

## Treatment

According to the Society of Clinical Child Adolescent Psychology (2022), Division 53 of the American Psychological Association, the only treatment that works well for anxiety disorders with children under 8 years of age is family-based cognitive behavioral therapy (CBT). With older children and adolescents some behavioral therapy techniques (exposure, modeling) and various modalities of CBT (family, with parents, combined with drugs) have proven to be effective. For this reason the articles focus on this model of therapy.

The article by Bertie and Hudson is a brief review of individualized CBT interventions. The authors discuss narrative, systematic, and meta-analytic reviews on the topic, present a model to describe the state of research, and a research agenda to advance the field. The transdiagnostic approach is justified by the high rate of comorbidity of internalizing problems, including anxiety and depression. Fujisato et al. conduct the Japanese adaptation of the Unified Protocol for Transdiagnostic Treatment of Emotional Disorders in Children (UP-C) and conduct a promising pilot study in children with a primary diagnosis of anxiety, obsessive-compulsive or depressive disorders to analyze the feasibility and efficacy of the protocol.

A couple of articles address problems with CBT. Alenezi et al. report that cognitive behavioral therapy is underutilized in clinical practice, despite being the first-line treatment for numerous disorders. They suggest that this phenomenon may be due, among other factors, to poor knowledge and negative parental attitudes toward CBT, so they conducted a study to assess these variables in parents of children with anxiety problems. Social anxiety disorder is one of the anxiety disorders that least responds to CBT, considered the treatment of choice. Consequently, Carlton et al. review the potential of mindfulness-based interventions, an under-explored therapeutic alternative in the adolescent population compared to the adult population.

This section also includes a theoretical proposal. Ingul et al. present the ECHO conceptual model to evaluate preventive interventions for emotional problems in children. It is an attempt to overcome the limitations of traditional randomized controlled trials, which report globally whether the intervention is effective, but do not clarify which components are responsible for the change.

## Final Words

The world seems to have gone mad. Environmental degradation, climate change, migratory movements, economic crises, citizen insecurity, street violence, terrorist attacks or armed conflicts are widespread concerns among citizens. Alongside these threats, there are other serious health risks such as AIDS, drugs, traffic accidents and pandemics. Children and adolescents also have their own concerns, such as mistreatment, sexual abuse, school failure, bullying and interpersonal difficulties. Already at the turn of the millennium, Twenge (2000) warned that we live in the “age of anxiety,” and childhood is no exception.

We wish to express our most sincere and deepest gratitude to the authors who have collaborated in this Research Topic. We are convinced that their contributions will promote the advancement of knowledge on psychopathology, assessment and treatment of emotional problems and anxiety disorders in childhood, which is a grain of sand in the construction of a better world, because the children of today are the men and women of tomorrow.

## Author Contributions

FM wrote a draft of this editorial. All authors reviewed, included comments, and approved the draft.

## Conflict of Interest

The authors declare that the research was conducted in the absence of any commercial or financial relationships that could be construed as a potential conflict of interest.

## Publisher's Note

All claims expressed in this article are solely those of the authors and do not necessarily represent those of their affiliated organizations, or those of the publisher, the editors and the reviewers. Any product that may be evaluated in this article, or claim that may be made by its manufacturer, is not guaranteed or endorsed by the publisher.
